# Death in the Emergency Department: A Retrospective Analysis of Mortality in a Swiss University Hospital

**DOI:** 10.1155/2019/5263521

**Published:** 2019-09-02

**Authors:** Eric P. Heymann, Alexandre Wicky, Pierre-Nicolas Carron, Aristomenis K. Exadaktylos

**Affiliations:** ^1^Department of Emergency Medicine, Neuchatel Cantonal Hospital (HNE), Neuchatel 2000, Switzerland; ^2^Department of Emergency Medicine, Lausanne University Hospital (CHUV), Lausanne 1005, Switzerland; ^3^Department of Emergency Medicine, Bern University Hospital (Inselspital), 3010 Bern, Switzerland

## Abstract

Acute treatment in emergency medicine revolves around the management and stabilization of sick patients, followed by a transfer to the relevant medical specialist, be it outpatient or inpatient. However, when patients are too sick to be stabilized, i.e., when the care provided in the Emergency Department (ED) may not be sufficient to enable transfer, death may occur. This aspect of emergency medicine is often overlooked, and very few public data exist regarding who dies in the ED. The following retrospective analysis of the mortality figures of a Swiss university hospital from January 1st 2013 to December 31st 2016 attests to the fact that with an incidence of 2.6/1,000, death does occur in the ED. With a broad range of aetiologies, clinical severity at presentation has a high correlation with mortality, a finding that reinforces the necessity of good triage system. Our analysis goes on to show that however (in)frequent death in the ED may be, there exists a lack of advanced directives in a majority of patients (present in only 14.8% of patients during the time of study), a worrying and often challenging situation for Emergency Medicine (EM) teams faced with premorbid patients. Furthermore, a lack of such directives may hinder access to palliative care, as witnessed in part by the fact that palliative measures were only started in 16.6% of patients during the study. The authors hope this study will serve as a stepping stone to promote further research and discussion into early identification methods for patients at risk of death in the ED, as well as motivate a discussion into the integration of palliative care within the ED and EM training curriculum.

## 1. Introduction

The increasing use of Emergency Departments as the first point of contact for healthcare is a multifactorial issue that is present on a global scale [1, 2]. Associated with an increased risk of mortality in the Emergency Department (ED), this phenomenon challenges us to the question: how are we as Emergency Physicians preparing to care for these (sometimes inevitable) deaths? And what measures do we have in place in our EDs to improve end-of-life care? From chronic disease to acute rapidly progressing pathologies, patients identified as dying (or palliative) were once rapidly transferred out of the ED to a more appropriate setting for end-of-life, yet as healthcare systems reach near saturation on a quasi-permanent basis [3, 4], once rapid transfers are now no longer possible and as patient time in the ED increases, so does the likelihood of mortality within the department. Switzerland has not been spared by this trend. Many measures have been taken to improve early identification of those requiring rapid transfer out of the ED (such as continuously revised triage scores [5]), yet these may not suffice in light of sheer increases in patient numbers and increases the chance of mortality within the ED. More commonly discussed behind private doors through mortality audits and rarely mentioned in the medical literature, we sought to open our database of mortality figures to the scientific community in an effort to promote further study of this often nil-discussed aspect of acute care and help establish a basis for which further studies can use to compare. The following retrospective study is an analysis of the mortality figures from a Swiss university hospital emergency between January 1st 2013 and December 31st 2016. Our data will be presented according to a standardized descriptive approach using nonclinical and clinical traits, with an additional secondary discussion on the potential to “predict death in the ED.” We will conclude our paper on the potential palliative measures that can be started in the Emergency Department.

## 2. Setting

**Figure 1:**
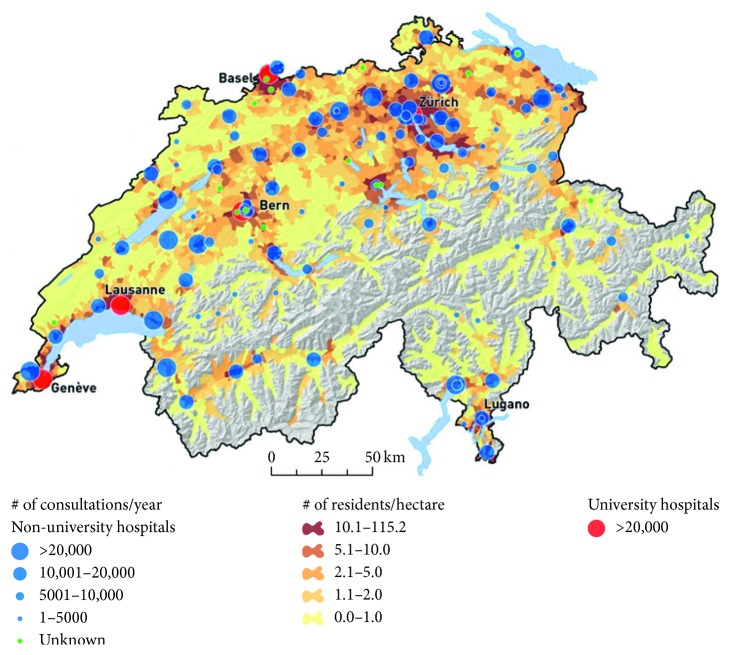
Hospital distribution in Switzerland.

The Bern University Hospital (Inselspital) is located in the capital city of Switzerland, in a bilingual but predominantly Swiss-German area (see Figure 1). It serves as a primary (city of Bern), secondary (canton of Bern), and tertiary centre for both local and international referrals. The hospital offers 24/7 support for all specialties and is one of the five trauma centres in Switzerland. In 2018, the Inselspital Emergency Department saw 49,800 patients over the age of 16 (paediatric patients are seen in a separate dedicated ED, on site). Patients are seen in one of the four mixed-speciality clinical areas: resuscitation (3 beds), inpatient and outpatient (24 beds), a fast-track outpatient consultation room (2 consultation rooms), and an observation unit (ED-CCU: 8 beds).

In accordance with the managed-competition liberal principles of Switzerland, the Swiss healthcare system functions on a premium-funded private insurance system, where competing insurers provide various health insurance plans, with a universal minimum set by the State and additional benefits depending on the premium paid [6]. All Swiss citizens are required to purchase a universal healthcare coverage plan. The provision of healthcare is ensured by a mixture of private and public actors, both within and outside the hospital setting (general practitioners, home follow-up nurses, elderly care, palliative care home teams, etc.). Standards are set on a federal level by the Confederation (Swiss Government).

## 3. Methodology

This monocentric retrospective analysis was conducted for patients seen between January 1st 2013 and December 31st 2016. The data from each patient who had died within the Emergency Department during the time of study were identified and extracted from the Electronic Medical Record (EMR) systems used in our ED (E-Care® and i-pdos®). Results were coded into a Microsoft Excel database by a dedicated data analyst. In conformity with current data privacy and Swiss Ethics Committee standards, once collected, these data were anonymised and all traceable (patient) information was removed from the sample; each patient was given a secondary randomised computer-generated identification number. A secondary “patient information” document linking the randomised number with the nominative patient information was created, password protected, and left unopened for the remainder of the study (to be used only in case of suspected data discrepancies, which for our study did not occur). From these data, an initial analysis was done to identify commonly available (in >95% of patients, when possible) clinical and paraclinical information; a secondary analysis was then performed on these isolated data. All data were analysed using Stata®.

A comprehensive review of the available literature was first conducted on June 1st 2017 using keywords “*Mortality*,*” “Death,*” “*Emergency Department,*” “*Emergency Room,”* “*Accident and Emergency,*” and secondary abbreviations (*ED*, *ER*, *A&E*). Of the literature identified from Pubmed online, Google Scholar, and Web of Science, we identified 7 studies that discussed mortality within the ED [7–13]. We further identified another 13 studies [14–26] which indirectly discussed mortality in the ED though not as a primary endpoint. In light of the relatively few studies identified, and in order to maximise comparative analysis, we repeated this literature review at the end of our data analysis on December 12th 2018, to include any new studies.

This study was approved by the Inselspital Ethics Board as well as the Bern subsection of the Swiss Ethics Committee (study ID number 2016-01531).

## 4. Outcome

105,001 patients over the age of 16 were seen in our Emergency Department between January 1st 2013 and December 31st 2016. During that period, a total of 277 patients died within our department, a mortality rate of 2.6/1,000. Of the 277 patients identified, 5 were excluded from our retrospective data analysis due to lack of data and 1 additional patient was excluded as seen in the paediatric ED and mistriaged in our EMR as an adult patient.

**Figure 2:**
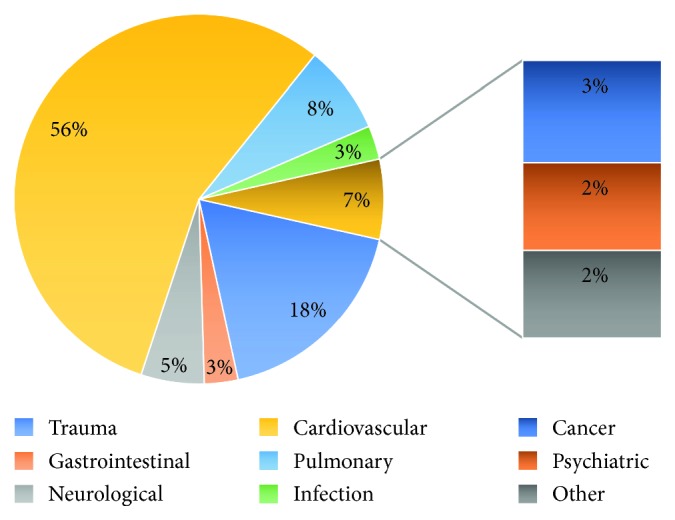
Pathophysiological classification of mortality.

In order to enable further subgroup analysis, the outcomes presented below include total (“overall”) figures as well as aetiologically subdivided results, based on the pathophysiological cause of death as established on the death certificate (see Figure 2).

### 4.1. Patient Characteristics

The mean mortality age was 65.7 years with a 2 : 3 female to male ratio. Of these, only 10.3% (28 patients) had known help at home, be it from a specialist carer or family. Advanced directives were known in only 14.8% of patients (40 of 271); 17.5% of those with known directives had proresuscitation instructions established. 13.8% of patients who died in our emergency during the period of study were of known foreign (“non-Swiss”) nationality.  Table 1 summarizes the patient characteristics of the 271 patients who died in our emergency department during the study period.

### 4.2. Triage

93.4% (*n* = 253) of patients who died during the study period were triaged as category 1 using the revised Swiss Emergency Triage Scale (rSET), i.e., requiring immediate medical attention. Subdivided into medical, surgical, or neurological field according to main presenting complaint (respectively, 74.1%, 22.9% and 3%), a large majority (82.3%) were initial directed and first seen in our resus bays (see Table 2).

Of the 271 patients who died in our Emergency Department during the study period, 65.7% were brought in as primaries, i.e., directly from scene, by Emergency Medical Services (EMS); an additional 18.1% were brought in by a physician-led EMS team. Family doctors and other hospital were responsible for 4.4% and 18% of admissions, respectively.  Table 3 summarizes these referral characteristics.

### 4.3. Clinical Parameters of Patients

The mean presenting GCS was 5.5 in all patients confounded, with a median value of 3.1. Only 23.3% of patients who died during the study presented to our ED spontaneously breathing without any mechanical assistance, the remainder 76.7% of patients already having been intubated; no patient under NIV at presentation died during their ED stay. Capillary refill time was >2 seconds in 80% of patients with available recorded values (in the EMR). 75% of patient brought in had unrecordable (initial) heart rates and (systolic) blood pressure due to being under CPR upon arrival.  Table 4 summarizes the clinical parameters of patients taken upon arrival (NB: due to the amount of data for this section, the (category) axes have been inverted).

### 4.4. Palliative Care and Predictability of Death

Palliative care, such as defined by the World Health Organisation, was started for 16.7% of patients during our study period. A careful review of the information available at triage from the authors and all senior clinical EM physicians revealed that 82.7% of deaths were predictable (see Table 5).

## 5. Discussion

The limited number of comparative studies dedicated to the analysis of mortality within the emergency department meant the authors had “free reign” to decide what variables to study. As such, upon presentation of our initially extracted patient data sets, an analysis was done to identify common variables, i.e., available to us for the majority of patients, to enable an appropriate comparative analysis. When possible, we set a minimum of 95% of patients having these variables, unless we deemed the information to be of interest for future comparative studies (discussed below). Furthermore, and to enable subgroup analyses, we subdivided our patient population according to the pathophysiological cause of death as reported on the certificate of death (see Figure 2). Contrary to many other studies available in the literature, the focus of our analysis was not to determine whether the reported diagnosis correlated with post-mortem diagnosis, though we did try to integrate any postmortem results that were available (post-mortems were done during the studied period—post-mortems are now becoming the norm for all ED deaths as an educational/continuous feedback tool).

With a mortality rate of 2.6/1,000 patients, our study reflected that of other available studies [7, 8, 27]. Though not reflective of the general population, this figure confirms what we already suspected that ED tends to the more vulnerable and sick; thus it comes as no surprise that the mean mortality age during the study period was 65.7 years, a figure also seen in the few other available comparative studies [8, 10–13].

Cardiovascular pathologies represented more than half of the causes of death, a figure which correlates with the Swiss statistics for 2015 [28] as well as European figures [29]. Surprisingly, in comparison to other national statistics, the second cause of mortality in our ED was trauma, a finding that can be explained by the geography of Bern in relation to Switzerland and the Alps (see map of Switzerland, Figure 1). Interestingly, this subgroup also had the highest percentage of foreigners (in relative terms, 30.8% vs 13.7% for cardiovascular deaths, the variable with the next highest number of foreigners). We explain this finding by the fact that our ED is the main trauma centre for the central Alps, home to most of the famous “pics” of the Swiss mountainscape, a preferred destination for tourism. When planning our study, we had initially opted to study a “nationality” variable as our ED sees a large number of foreign nationals every year, both tourists and residents (in 2015, according to the Swiss Federal Statistical Office, 24.9% of its population were non-Swiss nationals), and were thus not surprised to find that nearly 13.8% of all deaths occurring at our ED were of foreign individuals. Comparison with overall figures was not possible as nationality was not automatically integrated within our EMR for the 105,001 patients seen during the study period (nationality is on the other hand mandatory to establish a death certificate). We have now integrated this in our EMR to enable further statistical studies.

Living conditions for those presenting to an ED are important public health information: with regular contact with the vulnerable, EDs can serve as a gateway to open more social support services for patients in need. Unfortunately, the retrospective nature of our paper and the subject studied meant we could not obtain information on living conditions for those included in our study, and as such 85.2% of patients did not have this information recorded in their EMR. We have identified as a failure on our behalf (could this have helped prevent deaths in the long term?) and have now included this in a departmental policy, where this information is systematically collected in our EMR, and social services teams are at hand to help and assist patients identified as potentially vulnerable, in a manner similar to what the PREDICT score has done for advanced care planning [30].

As witnessed by the numerous discussions pertaining to the matter in the available medical literature, and a common trait in many societies, discussing end-of-life care and advanced directives holds a certain taboo in Switzerland (we define advanced directives as “what to do/not do if…” including CPR) [31], and we were surprised that in a country where assisted suicide is legal and openly discussed, only 14.8% of patients had known directives (see Table 1). The Federal Office of Public Health [32] as well as the Swiss Academy of Medical Sciences and the Swiss Medical Association [33] had previously identified this as a public health issue and had begun addressing this issue through widespread advertisements, the development of apps and the creation of an online (federal) registry made available to patients and shared with healthcare centres. We have now begun to address this as an institution by having end-of-life care discussions with all admitted patients and recording this in our EMR (updated if the patient changes their mind).

Patients admitted to our emergency department during the study period were all triaged using the revised Swiss Emergency Triage Scale (rSET), an externally validated and well-known triage algorithm used in many French speaking countries [5]. Patients are divided into four categories depending on severity of presenting complaint, and each category corresponds to a degree of urgency (with 1 being the highest level, or “immediate attention needed”). Triage is done for all walk-in patients, as well as for patients “brought in” by land or air ambulance via direct communication with the EMS team. 93.3% of those deceased had been given a triage score of 1, which suggests both a low rate of undertriage and a good sensitivity of our triage tool our ED. Reliability of our triage tool was also reinforced by the fact that 82.7% of patients were seen in one of our three resus bays.

An important part of patient flux and a key feature in lean is understanding what referral systems are in place and from where your patients originate. To understand this aspect of ED organisation, we included a “referred by” category to our data extraction. EMS (via dispatch, from scene straight to regional tertiary hospital) and physician-staffed ambulances (both land and air) accounted from 83.8% of all referrals (65.7% and 18.1%, respectively). The tertiary aspect of our hospital further explains why 11.4% were referred from another hospital, whereas the quite low percentage of patients who self-referred to our ED (*n* = 1, 0.4%) can be explained by the fact our hospital is located slight outside the city, an intentional purpose to allow smaller EDs within the city to first see and triage low-risk patients.

Our emergency department still operates under a traditional “specialist-led” format though this is now becoming obsolete as our teams are now polydisciplinary and Emergency Medicine is gaining recognition as an independent speciality in Switzerland (Switzerland still holds no valid Emergency Medicine (EM) specialist title, a fact due in part to political pressure from other specialities). Nevertheless, we have included this category as a bid to allow further comparative studies for countries where this old-system still prevails and EM is not yet recognised as a fully-fledged specialty (though this trend is also rapidly fading).

The retrospective nature of our study limited the amount of data that could be used as very few clinical parameters were available in sufficient numbers (of patients) to allow comparison. We nevertheless included curtain parameters even if they were not present in >95% of patients, as these clinical parameters are believed to be a good reflection of the patient's condition (as witnessed by their use in many clinical scores, include SIRS and qSOFA). The values correspond to those taken upon triage/arrival in the ED.

Although subject to controversy, the Glasgow Coma Scale (GCS) score is widely recognised as a clinical indicator of overall neurological deficit [3–7, 14–21]. The mean GCS of 5.5, with a median of 3.1, is a good reflection of the condition our patients arrived at in our ED (we opted for a mean and median rather than detailed/categorised GCS score (eyes/verbal/motor) to give an overall impression). A range and standard deviation were also calculated and provided no additional information; hence, only the former two parameters were included in the final analysis.

Witness to the critical condition of the deceased, 76.7% of patients who died within our ED were already intubated upon arrival, the remainder of patients spontaneously breathing. Noninvasive Ventilation was not seen in any patients, a feature which can be explained by the late adoption of NIV by our prehospital EMS teams. The retrospective nature of this study unfortunately does not allow us to compare these figures to the number of intubated patients who survived during the study period, nor does it allow us to determine why these patients were intubated in the first place.

In light of the above as well as in view of the fact that 72% of patients dying in our ED were undergoing cardiopulmonary rescucitation (CPR), we suspect that the 76.7% of patients with a Capillary Refill Time (CRT) > 2 s can be explained in part by the good quality of CPR (and may generate interest for future studies: can CRT be used as a clinical marker in patients undergoing CPR?).

Palliative care has been identified in the literature as an area where EDs fall short of their role and responsibilities towards patients [34, 35]. For the purposes of this study, the authors used the World Health Organisation's definition of palliative care: “*an approach that improves the quality of life of patients and their families facing the problem associated with life-threatening illness*, *through the prevention and relief of suffering by means of early identification and impeccable assessment and treatment of pain and other problems*, *physical*, *psychosocial and spiritual.”* The authors further accept that Palliative Care does not automatically assume that the outcome is death, and many patients on palliative care are later taken off as the underlying pathologies improve. Nonetheless, in light of the relatively short time patients are cared for by the ED, an involuntary association between predicted death and palliative care can be seen. We identified 45 cases where the healthcare team identified that the patient was dying and introduced additional measures that corresponded to the above definition of palliative care; these measures included the administration of medications as prescribed by the senior attending physician on call, as per protocol for our ED. Measures such as stopping CPR (underway in 72% of patients on arrival) were not considered palliation but simply clinical decisions based on internationally recognised prognostication criteria [14, 36, 37].

Of the 7 patients who passed away from complications of cancer, 6 benefited from some form of palliative care/accompaniment. It is important to bear in mind that this number may seem small but one should take in count that unless haemodynamically unstable or very ill, cancer patients are usually fast-tracked to a ward for treatment rather than spend time in the ED; this may further explain why only 2.6% of deaths for our study were attributed to an oncological aetiology.

The authors share the opinion of several other authors [34, 35, 38] that more training is required for EM physicians and that palliative care should be a component of the EM training curriculum and palliative care should be considered/started whenever a dire clinical outcome is predicted for a patient (see below).

To conclude this retrospective analysis, we included one subjective variable in our study: a yes/no answer to whether or not the death of the patient was predictable, given the information from the triage notes. This (very) subjective question will undoubtedly raise controversy (the “retrospectroscope” is always easier than the “prospectroscope”), but we believe that this question should be asked (and very often is, at least by senior physicians) in light of human suffering and the costs of healthcare associated with *nonsense* therapy. Each case was reviewed by the authors (>8 years of EM experience), and a simple question was asked: could the death be viewed “predictable” from the little information we had at triage. In 224, or 82.7% of cases, death seemed the likely outcome, mostly in patients under CPR or of advanced age.

Ideally, this “predictable death question” should be answered by a validated objective clinical scoring system, and we hope that by publishing our data, we will pave the way for future comparative studies that will enable the identification of variables that can be measured and correlate with clinical outcome, in a fashion similar to the PREDICT score (for example), which is validated for prediction of mortality at 1 year following ED visit (and thus promotes palliative care consultation and DNR discussion with the ED at time of consultation) [30].

## 6. Limitations

The authors identified many limitations to this monocentric study, monocentric being also being a limiting feature. While most other limitations were presented and integrated into the discussion above, we believe two key points should be further discussed.

The first limitation of this study concerns outcomes. When we initially set up this study we identified two key endpoints that we wanted to impart on the reader. Firstly, it was to provide a stepping stone for further studies (published and non-published, e.g. for hospital audits) to be able to compare their data with another ED, and we believe we have achieved this. Only through the publication of such data can we motivate further work (and discussion) into who dies in the ED, through the creation of ED mortality registries for example. This will hopefully also help generate in palliative care, and help provide the best form of care for end-of-life in the ED. The second endpoint we initially had aimed for was to identify key triage variables that could be used to develop a clinical score to predict ED mortality. Unfortunately, the retrospective nature of this work was a major obstacle to this, as we were often faced with incomplete triage information and had insufficient data common to a majority of patients. All the information we obtained in sufficient number is presented above.

The second limitation we believe should be mentioned is closely associated with the limitation of a retrospective study. When including variables to analyse, often times more information is better to help one understand said variables (such as the dynamic process behind indications and clinical decisions). This was a severely limiting factor for us (e.g.: how long was CPR done for before the patient was pronounced dead?). An aspect of this which we believe could have helped us better understand and prevent future deaths in our ED is determining the length of stay in the ED prior to death. Indeed, as mentioned previously, the traditional care pathway model for patients who had been identified as at risk of rapid (clinical) deterioration and death was to rapidly transfer these patients to a ward or hospice (if available) where a quieter setting is provided for end-of-life care. The current state of EDs worldwide and chronic saturation of hospital beds (see *Introduction*) is challenging this traditional care pathway model as flux is limited. One therefore has to ask oneself when reviewing the above data: how much of the patients who died during our study period did so because of “overstay” caused by ward/patient flux blockages/blockades. We cannot retrospectively extract this information, however we recommend that any further study into the matter of ED death include in its data a section on duration of ED stay prior to death, and where possible a section on time between when the patient could theoretically be transferred to a different section versus when the patient was actually transferred.

## 7. Conclusion

The above retrospective analysis presents the characteristics of mortality in our Emergency Department over a period of 4 years. It reflects the geo-ethnological characteristics as well as provides insight into the problems facing end-of-life care in a western European hospital. The authors have opted to publish these data in the hopes that it will provide a comparative baseline for others who wish to review their mortality figures in light of the rare availability of such information in the medical literature. We hope it will serve as a stepping stone for those wishing to study ED mortality and hopefully aide in developing a mortality predictability score for EDs. Furthermore, the authors hope these data will help challenge the reader to determining what place palliative care should have in emergency medicine and what can be done to improve end-of life care and accompaniment measures when death does occur in their emergency department.

## Figures and Tables

**Table 1:** Patient characteristics.

Aetiology (*n* = ; % of total)	Mean age (years)	Female(male)	Nationality			Help @ home				Advanced directives		
			Swiss	Foreign	N/A	Professional	Family	None	N/A	Yes	No	N/A
Trauma (49; 18.1%)	55.6	13 (36)	37	12	0	0	0	3	46	2	1	46
Gastrointestinal (8; 3.0%)	65.8	5 (3)	8	0	0	2	0	0	6	1	1	6
Neurological (15; 5.5%)	80	10 (5)	14	1	0	3	0	0	12	3	0	12
Cardiovascular (151; 55.7%)	69.5	43 (108)	131	18	2	5	6	1	139	10	4	137
Pulmonary (21; 7.7%)	69.9	7 (14)	19	2	0	2	1	1	17	5	0	16
Infection (8; 3.0%)	63.8	3 (5)	7	1	0	4	1	0	3	3	1	4
Cancer (7; 2.6%)	68.6	1 (6)	5	2	0	4	0	0	3	7	0	0
Psychiatric (6; 2.2%)	52.7	3 (3)	6	0	0	0	0	0	6	1	0	5
Other (6; 2.2%)	47.8	3 (3)	5	1	0	0	0	1	5	1	0	5
Total (271; 100%)	**63.7**	**88 (183)**	**232**	**37**	**2**	**20**	**8**	**6**	**237**	**33**	**7**	**231**

**Table 2:** Triage.

Aetiology (*n* = ; % of total)	Triage				Specialty		First seen in		
	1	2	3	4	Medicine	Surgery	Neurology	Resus	Acute care
Trauma (49; 18.1%)	48	1	0	0	6	43	0	47	2
Gastrointestinal (8; 3.0%)	6	2	0	0	6	2	0	4	4
Neurological (15; 5.5%)	12	2	1	0	6	2	7	9	6
Cardiovascular (151; 55.7%)	147	3	0	1	146	5	0	132	19
Pulmonary (21; 7.7%)	19	2	0	0	20	1	0	14	7
Infection (8; 3.0%)	7	0	1	0	6	1	1	4	4
Cancer (7; 2.6%)	2	1	3	1	5	2	0	3	4
Psychiatric (6; 2.2%)	6	0	0	0	2	4	0	5	1
Other (6; 2.2%)	6	0	0	0	4	2	0	6	0
Total (271; 100%)	**253**	**11**	**5**	**2**	**201**	**62**	**8**	**224**	**47**

**Table 3:** Referral characteristics.

Aetiology (*n* = ; % of total)	Referred by						
	Family doctor	EMS	Own initiative	Family member	Friend/colleague	Prehospitalretrieval doctor	Otherhospital
Trauma (49; 18.1%)	0	20	0	0	0	26	3
Gastrointestinal (8; 3.0%)	0	2	0	0	0	0	6
Neurological (15; 5.5%)	1	9	0	0	0	1	4
Cardiovascular (151; 55.7%)	5	116	1	0	0	17	12
Pulmonary (21; 7.7%)	3	10	0	0	0	4	4
Infection (8; 3.0%)	2	5	0	0	0	0	1
Cancer (7; 2.6%)	0	7	0	0	0	0	0
Psychiatric (6; 2.2%)	1	4	0	0	0	1	0
Other (6; 2.2%)	0	5	0	0	0	0	1
Total (271; 100%)	**12**	**178**	**1**	**0**	**0**	**49**	**31**

^*∗*^EMS = emergency medical services.

**Table 4:** Clinical parameters of patients.

		Trauma (49; 18.1%)	Gastrointestinal (8; 3.0%)	Neurological (15; 5.5%)	Cardiovascular (151; 55.7%)	Pulmonary (21; 7.7%)	Infection (8; 3.0%)	Cancer (7; 2.6%)	Psychiatric (6; 2.2%)	Other (6; 2.2%)	Total (271; 100%)
GCS	Mean	4.1	7.1	5.5	3.6	5.8	6	6.7	3.2	3	5.5
	Median	3	3	3	3	3	3	4	3	3	3.1

Airway	Intubated	34	4	7	132	15	4	1	5	6	**208**
	NIV	0	0	0	0	0	0	0	0	0	**0**
	Spontaneous breathing	15	4	8	19	6	4	6	1	0	**63**

CRT (in sec.)	<2	14	2	7	15	8	4	2	1	0	**53**
	>2	35	*5*	5	134	11	3	4	5	6	**208**
	N/A	0	*1*	3	2	2	1	1	0	0	**10**

Initial HR (/min)	<40	1	0	0	5	0	0	0	1	0	**7**
	40–60	2	0	0	3	0	0	1	0	0	**6**
	60–100	8	0	2	2	2	1	0	1	0	**16**
	100–120	7	3	3	3	1	0	1	0	0	**18**
	>120	4	0	3	4	2	4	2	0	0	**18**
	Under CPR	27	5	5	129	14	2	2	4	6	**195**
	N/A	0	0	2	5	2	1	1	0	0	**11**

Initial systolic BP (mmHg)	<90	10	1	0	11	2	1	2	2	0	**29**
	90–110	1	1	3	3	1	1	0	0	0	**10**
	110–140	4	0	2	1	0	2	1	0	0	**10**
	140–180	5	0	1	1	2	0	1	0	0	**10**
	>180	2	1	2	2	0	0	0	0	0	**7**
	Under CPR	27	5	5	129	14	3	2	4	6	**195**
	N/A	0	0	2	4	2	1	1	0	0	**10**

^*∗*^GCS = Glasgow Coma Scale; CRT = capillary refill time; HR = heart rate; BP = blood pressure.

**Table 5:** Palliative care and predictability of death.

Aetiology (*n* = ; % of total)	Palliative care started		Predictable death	
	Yes	No	Yes	No
Trauma (49; 18.1%)	8	41	43	6
Gastrointestinal (8; 3.0%)	2	6	5	3
Neurological (15; 5.5%)	7	8	11	4
Cardiovascular (151; 55.7%)	10	141	133	18
Pulmonary (21; 7.7%)	8	13	16	5
Infection (8; 3.0%)	3	5	1	7
Cancer (7; 2.6%)	6	1	6	1
Psychiatric (6; 2.2%)	0	6	6	0
Other (6; 2.2%)	1	5	3	3
Total (271; 100%)	**45**	**226**	**224**	**47**

## Data Availability

The data used to support the findings of this study are restricted by the Swiss Ethical Committees of Vaud and Bern, in order to protect patient privacy. Data are available from the corresponding author for researchers who meet the criteria for access to confidential data, after clearance from the Swiss Ethical Committees of Vaud and Bern.
